# Piezoelectric and conventional rotary techniques for mandibular impacted third molar extraction: A comparative study

**DOI:** 10.6026/973206300210699

**Published:** 2025-04-30

**Authors:** Kuldeep Pal, Swati Tiwari, Sumit Patidar, Rashmi Rai, Anurag Tripathi

**Affiliations:** 1 Department of Oral & Maxillofacial Surgery, Pal Hospital, Sagar, Madhya Pradesh, India; 2 Department of Oral & Maxillofacial Surgery, Dr. Mudgal's Sparsh Hospital and Research, Centre, Gwalior, Madhya Pradesh, India; 3 Department of Oral & Maxillofacial Surgery, Head & Neck Cancer, Reconstructive and Cranio-Maxillofacial Surgeon, Consultant at OraMax Clinic, Indore, Madhya Pradesh, India; 4 Department of Oral & Maxillofacial Surgeon, Balco Medical Centre, Raipur, Chhattisgarh, India; 5 Department of Oral & Maxillofacial Surgery, Clinic Oramax Dant Chikitsalaya, Bilaspur, Chhattisgarh, India

**Keywords:** Mandibular third molar, piezoelectric surgery, rotary instruments, postoperative pain, oral surgery, minimally invasive extraction

## Abstract

Surgical extraction of impacted lower third molars exists as a popular operation performed on the oral cavity. Piezoelectric surgery presents itself as an emerging minimal-invasiveness treatment option compared to traditional rotary instruments, which have been commonly used. Therefore, it is of interest to compare the clinical efficiency, postoperative outcomes and patient satisfaction between piezoelectric and conventional rotary techniques. The study evaluated two parameters: surgical time and postoperative pain, as measured by VAS scores, along with swelling severity, trismus development and healing rates during a 7-day assessment period. An extended period of surgery using piezoelectric techniques yields better post-operative outcomes than traditional rotary techniques, characterized by reduced pain levels and swelling, as well as improved functional restoration.

## Background:

The removal of mandibular third molars located beneath gum tissue continues to be the most common surgical procedure in oral and maxillofacial surgery. The standard procedure frequently results in several complications, including pain together with swelling trismus and delayed healing, so they negatively impact patient comfort during recovery [1, 2]. According to study reports, the conventional rotary hand-piece remains the preferred instrument for making osteotomy cuts and sectioning teeth during surgical procedures [3]. Conventional rotary devices produce elevated temperatures that create a mechanical impact on the nearby bones and soft tissues, which in turn raise the possibility of postoperative morbidity [4]. Scientists have developed piezoelectric surgery because this method applies ultrasonic micro-vibrations to precisely cut mineralized tissues with minimal damage to surrounding soft tissues [5, 6]. The procedure offers improved visual performance, minimal bleeding effects, and reduced surgical invasiveness, resulting in enhanced postoperative outcomes [7, 8]. A comparison of piezoelectric surgery with traditional rotary instruments, based on published research, has yielded inconclusive findings regarding procedural duration, while conflicting results have been reported on postoperative discomfort and the rate of surgical complications [9, 10]. Piezo devices require a longer duration for procedures, although they demonstrate advantages in lowering postoperative pain, as well as reducing soft tissue swelling and improving soft tissue recovery [11].

Progress in present-day surgical technology has compelled healthcare providers to adopt newer alternatives that protect tissues rather than relying on conventional methods. Piezoelectric surgery has become a widely adopted approach in third molar surgery and other dental procedures, including ridge splitting, sinus lifting, and bone harvesting, due to its precise delivery and safe operation [12]. Ultrasonic vibrations produce cavitation while maintaining blood-free irrigation, which creates both improved operative viewing and protects surrounding tissues from damage. These advantages are particularly valuable when treating anatomically challenging patients and cases with a higher risk of postoperative complications. The medical adoption of piezoelectric devices remains low due to their higher costs and the more extended training periods required for medical practitioners. Using piezosurgery extends the surgical duration to a level that becomes a critical operational consideration in dental clinics with high patient volumes [13]. Assessing postoperative advantages requires an investigation into their value when weighing them against increased operating time, as well as additional resource expenses [14]. Therefore, it is of interest to compare the clinical performance and postoperative outcomes of piezoelectric surgery with those of conventional rotary techniques in the removal of impacted mandibular third molars. The findings may help determine the more efficient and patient-friendly method in routine oral surgical practice.

## Materials and Methods:

The study analyzed 40 healthy individuals, ranging in age from 18 to 35 years, who required surgical extraction of their mesioangular impacted mandibular third molars. Forty participants received ethical approval from the institutional review board after undergoing selection at the outpatient department of oral and maxillofacial surgery, following their consent.

Participants were randomly divided into two equal groups (n=20 each) using a computer-generated randomization table:

[1] Group A (Rotary Group): Osteotomy and tooth sectioning were performed using a conventional low-speed handpiece with surgical burs.

[2] Group B (Piezoelectric Group): Osteotomy was carried out using a piezoelectric surgical unit equipped with bone-cutting inserts without damage to soft tissues.

The entire surgical procedure happened with local anesthesia (2% lidocaine added with 1:80,000 epinephrines) under the guidance of one proficient surgeon who wanted to reduce personal variation. A standard Ward's incision served as the initial procedure, followed by mucoperiosteal flap reflection in both surgical groups. The assigned techniques provided the research groups with proper procedures for bone removal and tooth sectioning. The healing process for all patients included continuous exposure to antibacterial medications (amoxicillin 500 mg, three times daily, for 5 days) and administration of a non-opioid analgesic (ibuprofen 400 mg, three times daily, for 3 days). Patients were instructed to apply ice packs to their faces for 24 hours after surgery. The clinical staff taught standard postoperative guidance to patients who received follow-up care on days 1, 3 and 7.

The research examined four important clinical assessment points:

[1] The entire surgical period was timed from the initial incision to the final suture placement using a stopwatch.

[2] The assessment of postoperative pain was conducted using the Visual Analog Scale (VAS) on days 1, 3 and 7.

[3] Facial swelling measurements included three-point measurements between the tragus and pogonion, from gonion to both lateral canthi and from gonion to the commissure.

[4] Conversion during mouth opening is measured by evaluating interincisal distance using a caliper.

[5] Medical personnel performed a clinical examination of wound healing, along with an assessment of potential complications, on day 7.

All data were entered into the SPSS version 25 software for statistical processing. The research design employed the Student's t-test, along with the chi-square test, to evaluate mean values and categorical differences. Statistical significance was indicated when a p-value reached a value of less than 0.05.

## Results:

The conducted research examined and contrasted the medical results between conventional rotary instruments and piezoelectric devices as techniques for extracting impacted mandibular third molars. Every participant finished the research without encountering any severe complications or withdrawals throughout the study. Patients in Group B who received piezoelectric treatment experienced more prolonged surgical procedures than those in Group A, who underwent rotary instrument procedures. The operating times for Group A averaged 24.6 ± 3.4 minutes, whereas Group B required 37.9 ± 4.1 minutes (p < 0.001), as shown in Table 1. The post-surgical pain measurements from the rotary group exceeded those of the rotary group during the entire healing period, as evaluated through Visual Analog Scale scores. Group A participants showed a mean VAS score of 6.2 ± 0.8 on day 1, which later decreased to 2.7 ± 0.6 by day 7. The patients in Group B experienced lower pain scores than those in Group A during the entire postoperative period, as indicated by the assessment results presented in Table 2. The dimensions of facial swellings expanded more extensively in Group A compared to Group B, as indicated by linear facial measurements. On day 3, Group A patients experienced an average tissue swelling of 2.4 ± 0.5 cm, whereas patients in Group B showed significantly lower swelling amounts at 1.3 ± 0.4 cm (p < 0.01) (Table 3). People in Group A had a more restricted mouth-opening capacity compared to those in Group B, as indicated by their interincisal measurement results. Group B patients experienced a more excellent mean interincisal opening of 32.4 ± 1.7 mm on day 3 than Group A patients, who measured 26.8 ± 2.1 mm (Table 4), confirming improved facial muscle comfort in the piezo group (Table 4). The piezo therapy resulted in superior wound healing on day 7, as 85% of treated patients achieved complete healing, compared to 60% in the rotary group (p = 0.04) (Table 5).

## Discussion:

This study evaluated the performance and healing outcomes of piezoelectric and conventional rotary tools used for the surgical extraction of impacted mandibular third molars. The piezoelectric method needed longer procedure times yet provided better healing results and less postoperative discomfort, including decreased swelling and trismus, when compared to the rotary approach. Previous examinations have shown identical surgical lengths in piezoelectric operations, as the ultrasonic tips maintain a conservative bone-cutting pace [1, 2]. Disadvantage of prolonged surgical time with piezosurgery remains tolerable, given that it causes minimal bleeding during surgery while, providing superior operative visibility [3, 4]. Postoperative pain was lower in the piezosurgery patients compared to the rotary group. It was found that the piezoelectric osteotomy produces less vibration and thermal injury, resulting in reduced nociceptor activation [5, 6]. Through micro vibrations during piezosurgery, the risks of inferior alveolar nerve and lingual nerve damage decrease substantially, thereby aiding in patient comfort [7, 8]. The post-surgical swelling together with trismus results directly from surgical trauma combined with inflammation. This research revealed reduced parameters in the piezo group, which supports earlier findings that ultrasonic bone-cutting methods produce less mechanical reaction within tissues [9, 10-11]. A detailed focus on this situation becomes crucial when extensive flap reflection coupled with bone removal exists because excessive tissue trauma can worsen postoperative issues [12].

Patients in the piezosurgery group exhibited better tissue repair compared to those in the conventional cutting technique group. Researchers attribute the benefits observed in the piezoelectric group to the piezoelectric devices' cavity formation mechanism and ultra-weak cutting sizes, which help retain and protect bone structure and blood vessel quality [13, 14, 15-16]. Research studies confirm that ultrasonic bone cutting leads to reduced necrosis of bone tissue while simultaneously preserving its vital osteocyte cells [17, 18]. Surgical duration increases somewhat, but practitioners managing multiple patients must consider the sustained benefits of piezosurgery treatment, especially regarding reduced patient recovery time, fewer complications and enhanced patient satisfaction [19, 20]. Training sessions and experience development can reduce the learning curve associated with piezoelectric devices, thereby shortening the procedure time in the future [21]. Piezosurgery exhibits improved safety characteristics, making it an optimal choice for both high-risk medical procedures and operations that require precision near anatomical critical structures [22]. The faster operating speed of conventional rotary instruments can result in unintended damage to vital soft tissues due to their broad cutting pattern [23, 24]. Available studies confirm that piezosurgery delivers better postoperative comfort and reduces complications, although it requires longer operating time [25, 26]. The various benefits of piezoelectric devices establish them as essential equipment for contemporary oral surgical practices, mainly when patient welfare is the primary concern.

## Conclusion:

Operation time is extended during piezoelectric surgical procedures compared to conventional rotary methods, but patients still receive critical clinical benefits. The clinical benefits of piezosurgery include lower discomfort levels, reduced swelling symptoms, and shorter trismus duration, as well as improved wound healing and enhanced patient comfort. The surgical removal of impacted mandibular third molars benefits from piezosurgery as a safe and innovative method that enhances patient care during this delicate procedure.

## Figures and Tables

**Table 1:** Comparison of mean surgical time between groups

**Group**	**Mean Time (minutes)**	**SD**	**p-value**
A (Rotary)	24.6	3.4	<0.001
B (Piezo)	37.9	4.1	

**Table 2:** Postoperative pain scores (VAS) over time

**Day**	**Group A (Rotary)**	**Group B (Piezo)**	**p-value**
1	6.2 ± 0.8	4.7 ± 0.6	0.002
3	4.3 ± 0.7	2.1 ± 0.5	<0.001
7	2.7 ± 0.6	1.3 ± 0.4	0.005

**Table 3:** Facial swelling (Mean increase in cm)

**Day 3**	**Group A (Rotary)**	**Group B (Piezo)**	**p-value**
	2.4 ± 0.5	1.3 ± 0.4	<0.01

**Table 4:** Interincisal opening (mm) - trismus assessment

**Day 3**	**Group A (Rotary)**	**Group B (Piezo)**	**p-value**
	26.8 ± 2.1	32.4 ± 1.7	0.003

**Table 5:** Interincisal opening (mm) - trismus assessment

**Healing Grade**	**Group A (n=20)**	**Group B (n=20)**	**p-value**
Complete Healing	12 (60%)	17 (85%)	0.04
Delayed Healing	8 (40%)	3 (15%)	
